# Incidence and Risk Factors of Childhood Pneumonia-Like Episodes in Biliran Island, Philippines—A Community-Based Study

**DOI:** 10.1371/journal.pone.0125009

**Published:** 2015-05-04

**Authors:** Hisato Kosai, Raita Tamaki, Mayuko Saito, Kentaro Tohma, Portia Parian Alday, Alvin Gue Tan, Marianette Tawat Inobaya, Akira Suzuki, Taro Kamigaki, Soccoro Lupisan, Veronica Tallo, Hitoshi Oshitani

**Affiliations:** 1 Department of Virology, Tohoku University Graduate School of Medicine, Sendai, Japan; 2 Research Institute for Tropical Medicine, Metro Manila,The Philippines; 3 Department of Pediatrics, Tohoku University Graduate School of Medicine, Sendai, Japan; University College London, UNITED KINGDOM

## Abstract

Pneumonia is a leading cause of deaths in infants and young children in developing countries, including the Philippines. However, data at the community level remains limited. Our study aimed to estimate incidence and mortality rates and to evaluate risk factors and health-seeking behavior for childhood pneumonia. A household level interview survey was conducted in Biliran Island, the Philippines. Caregivers were interviewed using a semi-structured questionnaire to check if children had symptoms suggesting pneumonia-like episodes from June 2011 to May 2012. Of 3,327 households visited in total, 3,302 (99.2%) agreed to participate, and 5,249 children less than 5 years of age were included in the study. Incidence rates of pneumonia-like episodes, severe pneumonia-like episodes, and pneumonia-associated mortality were 105, 61, and 0.9 per 1,000 person-years, respectively. History of asthma [hazard ratio (HR): 5.85, 95% confidence interval (CI): 4.83–7.08], low socioeconomic status (SES) (HR: 1.11, 95% CI: 1.02–1.20), and long travel time to the healthcare facility estimated by cost distance analysis (HR: 1.32, 95% CI: 1.09–1.61) were significantly associated with the occurrence of pneumonia-like episodes by the Cox proportional hazards model. For severe pneumonia-like episodes, a history of asthma (HR: 8.39, 95% CI: 6.54–10.77) and low SES (HR: 1.30, 95% CI: 1.17–1.45) were significant risk factors. Children who had a long travel time to the hospital were less likely to seek hospital care (Odds ratio: 0.32, 95% CI: 0.19–0.54) when they experienced severe pneumonia-like episodes. Incidence of pediatric pneumonia-like episodes was associated with a history of asthma, SES, and the travel time to healthcare facilities. Travel time was also identified as a strong indicator for health-seeking behavior. Improved access to healthcare facilities is important for early and effective management. Further studies are warranted to understand the causal relationship between asthma and pneumonia.

## Introduction

Pneumonia is one of the most important global health problems in children less than 5 years of age, especially in developing countries. The Integrated Management of Childhood Illness (IMCI), developed by the World Health Organization and the United Nations Children’s Fund, promotes better patient management in order to reduce morbidity and mortality resulting from common childhood diseases, including pneumonia. The Millennium Development Goal 4 (MDG 4) of the United Nations aims to achieve reduction in child mortality by two-thirds by 2015 from the 1990 level. However, many developing countries are not on track with achieving MDG4 [1]. Pneumonia is one of the factors hindering the achievement of MDG4, because it remains to cause 1.4 million deaths of children under the age of 5 years annually, accounting for 18% of overall mortality in this age group globally [2]. The estimated incidence rate of pneumonia in children less than 5 years of age in the Western Pacific region is 110 per 1,000 person-years [3]. The Philippines is a middle-low income country in the Western Pacific region. According to the annual reports from its Department of Health (DOH), pneumonia is ranked as the 1st leading cause of death in children aged 1–4 years [4].

Previous studies found a close association between childhood pneumonia and risk factors such as age, sex, parents’ educational level, parental smoking, number of siblings, type of cooking fuel, type of toilet facility, and socio economic status (SES) [5–12]. Notably, the aforementioned studies were conducted in hospital settings. When children develop pneumonia, caregivers may not always take them to hospitals, especially in countries with limited resources, because of various reasons, including financial constraints, lack of knowledge, and poor accessibility to healthcare facilities [13–18]. In order to understand the real situation of pediatric pneumonia, data on the healthcare-seeking behavior for pneumonia, particularly at the community level are required, but such data are still limited [12,19].

This study was designed to estimate the incidence rate and mortality of childhood pneumonia-like episodes and to evaluate the association between pneumonia-like episodes incidence and risk factors such as the socioeconomic status (SES) and travel time from the household to the healthcare facilities. The association between these factors and caregivers’ healthcare-seeking behavior was also analyzed. The findings of this study would be considered as the baseline information for a prospective cohort study, which has been started in the same area, as a part of the research project under Science and Technology Research Partnership for Sustainable Development.

## Methods

### Study population

**Fig 1:**
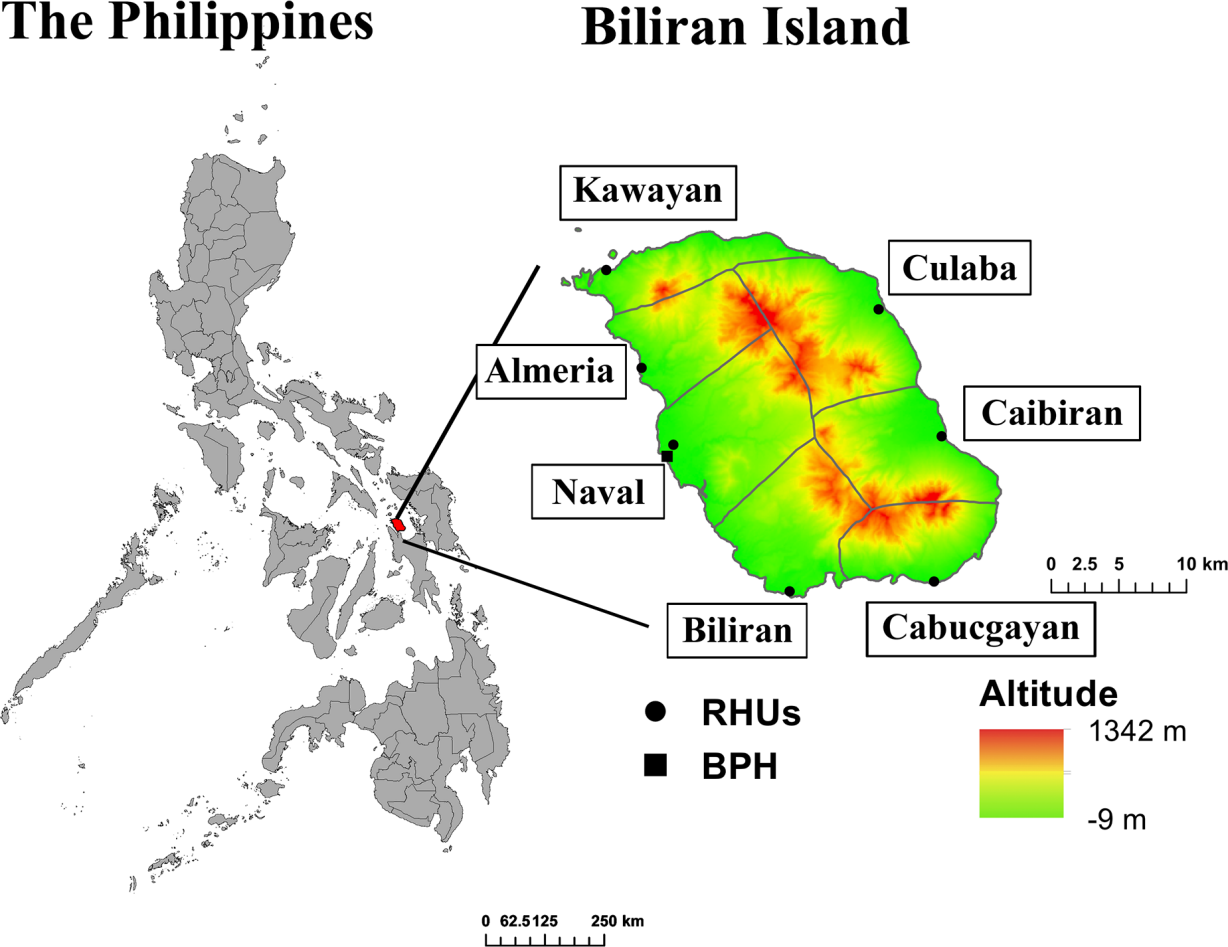
Study sites: Biliran Province, the Eastern Visayas Region (Region VIII), the Philippines. RHU: Rural health unit, Hospital: Biliran Provincial Hospital. Maripipi, municipality in a small island, is not included in the map.

A retrospective survey was conducted in Biliran Province in the Eastern Visayas region of the Philippines. Biliran Province consists of a main island (Biliran Island, 556 km^2^) and small islands, with a total of 161,760 inhabitants[20]. The province consists of eight municipalities, each of which is further divided into *barangays*. *Barangay* is the smallest administrative unit under municipalities in the Philippines, equivalent to a village in other countries. The study was conducted in seven of eight municipalities in Biliran Island (Fig 1). The seven municipalities included Naval (number of *barangays*: 26), Caibiran (17), Biliran (11), Cabucgayan (13), Kawayan (20), Culaba (17), and Almeria (13). One municipality, Maripipi, was excluded from the study because of its geographical location (isolated island) and small population size.

The public health system in the study area includes Barangay Health Worker, Barangay Health Station (BHS), Rural Health Unit (RHU), and Biliran Provincial Hospital (BPH). Barangay Health Workers are health-volunteers who assist in providing basic health services, including monitoring of children in the *barangay*. Each BHS is apportioned several *barangays* to which a midwife is assigned. BHSs provide pre-natal and post-natal care, health education, and public health services such as mass drug administration and immunization. A RHU is a primary healthcare facility located in each municipality to which a qualified doctor is assigned to provide outpatient care. Notably, medical consultation at the RHU is free of charge. Free medications for several common diseases are also provided depending on the availability and the stock. BPH, the only hospital in Biliran Province, is located in Naval, the capital city on the western coast of the island. BPH is a secondary level referral hospital consisting of departments such as Internal Medicine, Pediatrics, Obstetrics and Gynecology, and Surgery. It has a 75 bed-capacity with a bed-occupancy rate of 134% in 2011 [personal communication, hospital/other health facilities statistical report, 2011, DOH, Philippines]. Twenty beds are allocated to the pediatric ward. There were 443 children admitted to the Pediatric Ward of BPH because of pneumonia in 2011.

### Sample size

The target population was children less than 6 years of age. The population was estimated to be about 22,600 assuming that 14% of the total population of Biliran Island (161,760) belonged to this age category. The incidence of pneumonia in Biliran Island, based on data from local health officials, was estimated at 80 per 1,000 person-years. We calculated the household sample size with a marginal error of 2% with an alpha of 0.05 and power of 0.8, assuming that on average, one household has one child who is less than 5 years of age and the proportion of refusal for informed consent was 10%. Considering these margins, we decided to adopt a sample size of 3,300 households.

### Sampling methods

Cluster sampling was applied to select households. In each municipality, *barangays* were categorized into four types on the basis of their characteristics i.e., urban, rural-inland, rural-seaside, and both rural-seaside and-inland. Among 28 clusters (7 municipalities × 4 types of *barangays*), two-stage (*barangay* and household) systematic random sampling was applied and the sampling fraction (sample size/total population of children) in each cluster was kept constant as 0.020 (3,300/161,760). Using local health data, households that were reported to have children less than 4 years of age were visited.

### Data collection

A semi-structured questionnaire regarding demographic background, SES, and past medical history including recalled pneumonia-like episodes of under five children during June 2011 to May 2012 was used for the household survey. This retrospective survey by interview was conducted once for each household during June to August, 2012. The questionnaire was translated into the local languages Cebuano and Waray-waray. Trained local interviewers who were native speakers of these languages conducted the interview. Written informed consent was obtained from heads of households. In the case that a head of household was not a parent or guardian of the child participants, we obtained additional informed consent from a parent or legally authorized guardian. The geographic location of each household of the study participants, rural health unit, and Biliran Provincial Hospital was recorded using the geographic positioning system eTrex (Garmin International Inc., Olathe, Kansas). This study protocol was approved by the Ethics Committee of Tohoku University Graduate School of Medicine, Japan, and the Institutional Review Board of Research Institute for Tropical Medicine, the Philippines. This study had been conducted according to the principles described in the Declaration of Helsinki.

### Operational definition of pneumonia-like episode

If a child experienced a pneumonia-like episode from June 1, 2011 to May 31, 2012, further questions regarding the episode were asked. A pneumonia-like episode was defined based on symptoms recalled by caregivers, which included difficulty in breathing, cough and the inability to drink, or chest indrawing, together with fever. Among them, a severe pneumonia-like episode was defined as one with cough and the inability to drink or chest indrawing. We used the IMCI guidelines when defining our criteria, although we did not adopt them completely. We did not ask about stridor or wheezing because of the difficulty in explaining to or being understood by caregivers. We also did not ask about the respiratory rate because it was hardly recalled. To improve the specificity of the definition of a pneumonia-like episode, we included fever as one of the criteria [21]. We showed a video of chest indrawing to improve the caregiver’s understanding. A history of asthma was determined by asking caregivers if their children had been diagnosed with asthma.

If any children died at age of <5 years during the study period, verbal autopsy was performed by a specially trained interviewer. Pneumonia-associated deaths were identified by verbal autopsy using the aforementioned criteria set for pneumonia-like episodes.

### Social economic status score

A Simple Poverty Scorecard for the Philippines developed by Mark Schreiner was used to assess the SES score [22]. This scoring system was designed to assess SES of households in the Philippines by asking 10 simple questions (the number of children per household; school attendance of the child; female head’s education level; salaried employment; material of the house’s roof and walls; type of toilet facility; and possession of a refrigerator, television, or washing machine). Each question has a different score, so the weight of each item is different in the final SES score. The maximum total score is 100 and minimum is 0; a lower SES score indicates a lower SES.

### Cost distance analysis

Cost distance analysis was performed to estimate the travel time from each house to RHU or BPH by calculating the least accumulative cost-path across the smallest units of the map (raster) with data of the estimated base cost [23]. The base cost was defined by transportation methods (1: walking, if there is no road, 2: motorcycling if there is road, or 3: boating on the mangrove forest), slope (difference in the altitude by distance) [24], vegetation density for walking [25], and the speed of motorcycles depending on the types of the road (S1 Table). The land elevation map and road map were derived from the Shuttle Radar Topography Mission 90-m grid data and the OpenStreetMap, respectively, both of which were obtained from the PhilGIS website (http://philgis.org/. Accessed September 11, 2013). The land usage information derived from the satellite image from 2010 was obtained from the National Mapping and Resource Information Authority (NAMRIA, Manila), and vegetation levels were defined depending on the land use. The road map and the land-cover map, including the vegetation categories, were converted to the 90-m grid raster layers for calculation of the cost distance. The rasterization, cost distance calculation, and mapping was performed by the raster, gdistance, and maptools packages in R software (version 3.0.2, R Foundation for Statistical Computing, Vienna, downloaded from http://www.R-project.org). The cost distance data was included in the statistical models by four categories divided by quartiles.

### Statistical analysis

The incidences of pneumonia-like, severe pneumonia-like, and pneumonia-like-associated mortality were calculated per 1,000 person-years (S1 Dataset). We included children who were less than 5 years old from June 1, 2011 to May 31, 2012 when calculating person-years of children. Children who were ≥5 years old on June 1, 2011 or had not been born by May 31, 2012 were excluded from the analysis. Background characteristics among municipalities were compared using ANOVA, chi-square test, and Welch’s test using the JMP statistical software (version 10.0.2, SAS Institute Inc., Cary, NC.). The Cox proportional hazards model was used to estimate the hazard ratios of possible risk factors of pneumonia-like episodes or severe pneumonia-like episodes, with the background characteristics adjusted (S2 Dataset). Logistic regression models with generalized estimating equation [26] were used to evaluate the factors associated with healthcare-seeking behaviors, which included visiting any healthcare facility if pneumonia-like episodes occurred and visiting a hospital if severe pneumonia-like episodes occurred (S3 Dataset). Variables with a *p*-value of <0.1 in univariate analyses were included in a step-down procedure in multivariable models. The total SES score and individual factors included in the SES score were tested in separate multivariable models. A *p-*value of <0.05 was considered statistically significant. The Cox proportional hazards model and generalized estimating equation model were used in STATA 12 (Stata Corp., College Station, TX). Children who died within the study period were not included in the analysis but were included in the mortality rate calculation if they died from pneumonia-like episodes.

## Results

### Study participants

We visited 3,327 households, but informed consent could not be obtained from eight (0.2%). We thus excluded these eight households from our study without taking any data. Of the 5,342 children from the 3,319 households where interviews were conducted, we excluded 13 (0.2%) because of incomplete data and 80 (1.5%) because they were born after May 31, 2012 or had turned 5 years old by June 1, 2011. Finally, 5,249 (98.3%) children from 3,302 (99.2%) households contributed to 4,510 person-years of data. Of them, 3,612 (68.8%) children were counted as one person-year.

Table 1 shows the socio-demographic characteristics of the study participants. The mean age of participants was 2.9 years (SD: ±1.7). The proportion of boys and girls were similar (boys vs. girls: 51.1% vs. 48.9%, *p* > 0.5). Eight hundred and sixty-six children (16.6%) had a history of asthma diagnosis. The average SES score was 31.6 (SD: ±18.8). Age, preterm birth, and sex proportion were not significantly different among the seven municipalities, but other factors such as a history of asthma diagnosis, the average SES score, a household member with salaried employment, the type of toilet facility, the source of drinking water, and the possession of a washing machine were significantly different.

**Table 1:** Socio-demographics of study participants according to the municipality.

	Almeria	Biliran	Cabucgayan	Caibiran	Culaba	Kawayan	Naval	Total	*p*-value
**No. of households**	353	341	434	451	263	511	949	3302	
**No. of children less than 5 years of age**	518	525	746	762	428	762	1508	5249	
**Total person-years**	442	451	633	664	364	669	1287	4510	
**No. of pneumonia-like episodes**	35	37	80	93	25	69	135	474	
**No. of severe pneumonia-like episodes**	23	26	50	49	10	37	79	274	
No. of deaths*	1	1	0	0	2	0	0	4	
**Incidence rate of pneumonia-like episodes (per 1,000 person-year)**	79	82	126	140	69	103	105	105	
**Incidence rate of severe pneumonia-like episodes (per 1,000 person-year)**	52	58	79	74	27	55	61	61	
**Mortality (per 1,000 person-year)**	2.3	2.2	0.0	0.0	5.5	0.0	0.0	0.9	
**Mean age in years (SD)**	2.9 (1.7)	2.9 (1.7)	3.0 (1.7)	3.0 (1.7)	2.9 (1.7)	3.0 (1.6)	2.9 (1.7)	2.9 (1.7)	0.6905^a^
**Preterm gestational age at birth (%)**	6 (1.2)	7 (1.3)	11 (1.5)	7 (0.9)	5 (1.2)	13 (1.7)	28 (1.9)	77 (1.5)	0.6357^b^
**Sex, male (%)**	280 (54.1)	268 (51.2)	367 (49.3)	393 (51.6)	224 (52.3)	392 (51.4)	754 (50.0)	2678 (51.1)	0.6860^b^
**History of asthma (%)**	65 (12.6)	91 (17.6)	171 (23.1)	115 (15.2)	44 (10.4)	124 (16.3)	256 (17.0)	866 (16.6)	<.0001^b^
**Mean socioeconomic status score (SD)**	39.6 (19.5)	32.1 (17.6)	26.8 (17.1)	24.5 (15.6)	29.3 (16.9)	32.5 (18.4)	34.7 (20.2)	31.6 (18.8)	<.0001^a^
**Household member with salaried employment (%)**	243 (46.9)	150 (28.6)	159 (21.3)	140 (18.4)	88 (20.6)	243 (31.9)	906 (60.1)	1929 (36.8)	<.0001^b^
**Toilet, water sealed (%)**	400 (77.2)	321 (61.1)	375 (50.3)	309 (40.6)	234 (54.7)	461 (60.5)	920 (61.0)	3020 (57.5)	<.0001^b^
**Source of drinking water, treated water (%)**	412 (79.5)	501 (95.4)	711 (96.2)	714 (93.7)	382 (89.3)	622 (81.6)	1248 (82.9)	4590 (87.6)	<.0001^b^
**Having a washing machine (%)**	131 (25.3)	59 (11.2)	47 (6.3)	32 (4.2)	46 (10.8)	96 (12.6)	275 (18.2)	686 (13.1)	<.0001^b^
**Travel time (in hours) to the closest rural health unit or hospital (SD)**	0.096 (0.080)	0.099 (0.091)	0.107 (0.095)	0.122 (0.124)	0.075 (0.052)	0.176 (0.124)	0.140 (0.106)	0.124 (0.107)	<.0001^c^
**Travel time (in hours) to Biliran Provincial Hospital (SD)**	0.275 (0.086)	0.428 (0.079)	0.776 (0.132)	0.803 (0.077)	0.905 (0.064)	0.617 (0.163)	0.190 (0.128)	0.514 (0.293)	<.0001^c^

Incidence rate: number of episodes/1,000 person-years, Mortality: number of mortal events/1,000 person-years

a: ANOVA

b: Chi-square test

c: Welch’s test

*Number of deaths indicates the number of children who were suspected to have died of pneumonia-like episodes.

### Incidence rate of pneumonia-like episodes

The number of children who did not experience any pneumonia-like episode during the survey period was 4,843 (92.3%), and 406 (7.7%) children experienced pneumonia-like episodes once or more. Among 406 children, 474 pneumonia-like episodes had occurred, and 274 (57.8%) of pneumonia-like episodes were severe (Table 1). The incidence rate of pneumonia-like episodes was 105 per 1,000 person-years and that of severe pneumonia-like episodes was 61 per 1,000 person-years. The estimated annual number of pneumonia-like episodes in the entire Biliran Island was 1,976. Among seven municipalities, Caibiran had the highest incidence rate of pneumonia-like episodes (140 per 1,000 person-years) and Culaba had the lowest (69 per 1,000 person-years).

Among 406 children who experienced pneumonia-like episodes, 355 (87.4%) experienced one pneumonia-like episode, 45 (11.1%) experienced two episodes, and 6 (1.5%) experienced more than two episodes (S1 Fig). Difficulty in breathing, cough and the inability to drink, and chest indrawing were observed in 461 (97.3%), 144 (30.4%), and 207 (43.7%) children, respectively.

**Fig 2:**
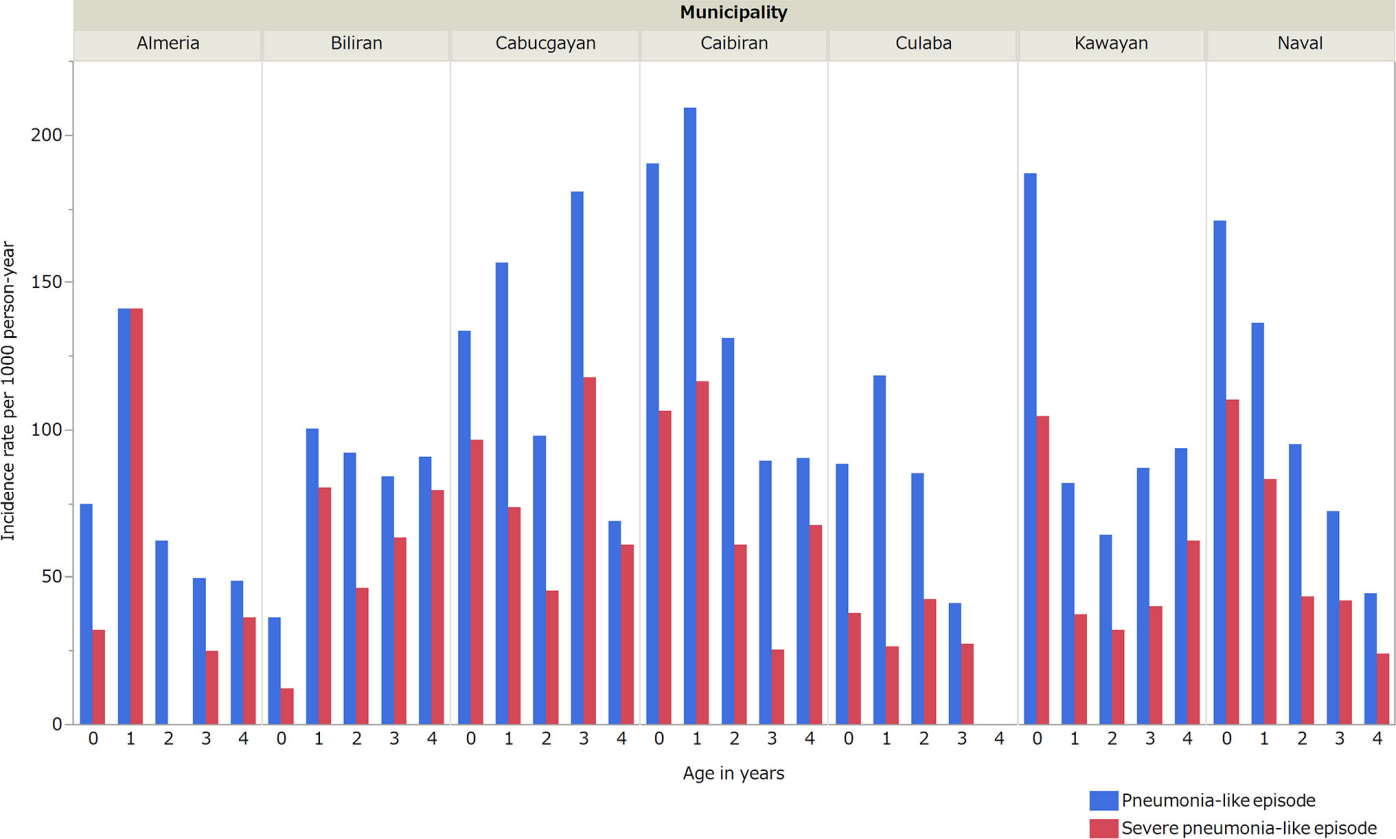
Incidence rates of identified pneumonia-like episodes in children less5 years of age according to the municipality and age in seven municipalities, Biliran, the Philippines.

Incidence rates by age groups varied between municipalities (Fig 2). More than half the pneumonia-like episodes (53.8%, 255/474) occurred in children aged <2 years.

### Risk factors of pneumonia-like episodes

The risk factors associated with the occurrence of pneumonia-like episodes and severe pneumonia-like episodes are listed in Tables 2 and 3. In multivariate analysis, lower SES [Hazard Ratio (HR): 1.11, 95% confidence interval (CI): 1.02–1.20], a preterm birth (HR: 1.87, 95% CI: 1.12–3.13), a history of asthma diagnosis (HR: 5.85, 95% CI: 4.83–7.08), drinking natural water or water from the well (HR: 1.44, 95% CI: 1.13–1.85), and a longer travel time to the closest medical facility (HR: 1.32, 95%: 1.09–1.61) yielded significant associations with the occurrence of pneumonia-like episodes. Children of an older age (HR: 0.74, 95% CI: 0.68–0.80), having a household member with salaried employment (HR: 0.80, 95% CI: 0.65–0.99), and having a washing machine (HR: 0.71, 95% CI: 0.51–0.99) were less likely to have experienced pneumonia-like episodes. With regard to severe pneumonia-like episodes, lower SES (HR: 1.30, 95% CI: 1.17–1.45), a history of asthma (HR: 8.39, 95% CI: 6.54–10.77), and drinking natural water or water from the well (HR: 1.87, 95% CI: 1.38–2.53) were significant risk factors. In multivariate analysis, children with an older age (HR: 0.73, 95% CI: 0.65–0.81) and who had a water-sealed toilet (HR: 0.57, 95% CI: 0.44–0.73) had a significantly reduced risk of experiencing severe pneumonia-like episodes. Factors, including parental smoking, the number of siblings, and the type of cooking fuel, were not significantly associated with the occurrence pneumonia-like episodes.

**Table 2:** Hazard ratios of each risk factor for pneumonia-like episodes in children less than 5 years of age in Biliran, the Philippines.

		Univariate			Adjusted		
		HR	95% CI	*p-*value	HR	95% CI	*p-*value
**Age of the child**	0	ref.			ref.		
**(in years)**	1	0.73	0.58–0.92	0.007	0.65	0.51–0.82	<0.001
	2	0.51	0.39–0.66	<0.001	0.44	0.33–0.58	<0.001
	3	0.57	0.44–0.74	<0.001	0.44	0.33–0.58	<0.001
	4	0.36	0.23–0.57	<0.001	0.28	0.17–0.45	<0.001
**Sex**	Female	ref.					
	Male	1.20	1.00–1.45	0.048			
**Caregiver's education**	<Secondary school	ref.					
**level**	≥Secondary school	0.83	0.68–1.00	0.046			
**Gestational age at birth**	Full term	ref.			ref.		
	Preterm	1.94	1.20–3.15	0.007	1.87	1.12–3.13	0.016
**History of asthma**	No	ref.			ref.		
	Yes	5.28	4.36–6.38	<0.001	5.85	4.83–7.08	<0.001
**Social economic status score**	1st quartile (highest)	ref.					
	2nd quartile	1.19	0.92–1.54	0.191	1.32	1.00–1.73	0.048
	3rd quartile	1.18	0.91–1.53	0.209	1.19	0.91–1.55	0.195
	4th quartile (lowest)	1.52	1.18–1.95	0.001	1.44	1.10–1.87	0.007
**Household member with salaried employment**	None	ref.			ref		
	≥One	0.74	0.61–0.91	0.003	0.80	0.65–0.99	0.040
**Having a washing machine**	No	ref.			ref.		
	Yes	0.68	0.49–0.94	0.018	0.71	0.51–0.99	0.037
**Having water-sealed toilet**	No	ref.					
	Yes	0.77	0.65–0.93	0.006			
**Having a refrigerator**	No	ref.					
	Yes	0.79	0.63–0.99	0.042			
**Having hard wall (concrete, clock, etc.)**	No	ref.					
	Yes	0.85	0.71–1.02	0.087			
**Source of drinking water**	Treated water	ref.			ref.		
	Well or natural	1.28	1.01–1.63	0.044	1.44	1.13–1.85	0.003
**Travel time to closest rural health unit or hospital**	<10 min	ref.			ref.		
	≥10 min	1.24	1.02–1.50	0.028	1.32	1.09–1.61	0.005
**Travel time to Biliran Provincial Hospital**	<30 min	ref.					
	≥30 min	1.23	1.02–1.48	0.027	1.21	1.00–1.46	0.054
**Municipality**	Almeria	ref.					
	Culaba	0.97	0.57–1.66	0.922			
	Biliran	1.15	0.70–1.90	0.579			
	Naval	1.40	0.93–2.10	0.105			
	Kawayan	1.42	0.92–2.19	0.113			
	Cabucgayan	1.73	1.13–2.66	0.013			
	Caibiran	1.86	1.21–2.84	0.004			

HR: Hazard ratio, 95% CI: 95% confident interval, socioeconomic status score: 1st quartile (41–92), 2nd quartile (27–40), 3rd quartile (16–26), and 4th quartile (0–15)

Educational system in the Philippines was preschool for three years (4–6 years old), elementary school for six years (7–12 years old) and secondary school for four years (13–16 years old).

The Cox proportional hazards model was used. Variables with *p*-value of <0.1 in univariate analyses were included in a step-down procedure in multivariable models. The total socioeconomic status score and the individual factors included in the socioeconomic status score were tested in separated multivariate models. A history of asthma, the source of drinking water, or geographic position of the house were missing in 12 episodes, 1 episode and 4 episodes, respectively; therefore, 457 episodes were included in multivariate analysis.

**Table 3:** Hazard ratios of each risk factor for severe pneumonia-like episodes in children less than 5 years of age in Biliran, the Philippines.

		Univariate			Adjusted		
		HR	95% CI	*p-value*	HR	95% CI	*p-value*
**Age of the child**	0	ref.			ref.		
**(in years)**	1	0.73	0.55–0.98	0.034	0.64	0.47–0.86	0.003
	2	0.44	0.29–0.64	<0.001	0.37	0.25–0.54	<0.001
	3	0.52	0.37–0.74	<0.001	0.37	0.26–0.52	<0.001
	4	0.49	0.29–0.83	0.007	0.38	0.22–0.65	<0.001
**Sex**	Female	ref.					
	Male	1.23	0.97–1.56	0.094			
**Caregiver's education level**	<Secondary School	ref.					
	≥Secondary School	0.63	0.49–0.82	0.001			
**Gestational age at birth**	Full term	ref.					
	Preterm	1.16	0.55–2.45	0.696			
**History of asthma**	No	ref.			ref.		
	Yes	7.49	5.81–9.66	<0.001	8.39	6.54–10.77	<0.001
**Social economic status score**	1st quartile (highest)	ref.					
	2nd quartile	1.17	0.80–1.70	0.415	1.43	0.97–2.09	0.070
	3rd quartile	1.55	1.10–2.19	0.012	1.71	1.19–2.43	0.003
	4th quartile (lowest)	2.19	1.57–3.05	<0.001	2.24	1.59–3.15	<0.001
**Household member with salaried employment**	None	ref.					
	≥One	0.70	0.53–0.92	0.010			
**Having a washing machine**	No	ref.					
	Yes	0.64	0.42–0.96	0.031			
**Having a water-sealed toilet**	No	ref.			ref.		
	Yes	0.61	0.48–0.78	<0.001	0.57	0.44–0.73	<0.001
**Having a refrigerator**	No	ref.					
	Yes	0.80	0.60–1.07	0.129			
**Having hard wall (concrete, block, etc.)**	No	ref.					
	Yes	0.68	0.53–0.87	0.002			
**Type of fuel for cooking**	Others	ref.					
	Firewood	1.53	1.08–2.16	0.015			
**Cooking place**	Outside	ref.					
	Inside with window	0.97	0.74–1.27	0.833			
	Inside without window	1.36	0.99–1.87	0.056			
**Source of drinking water**	Treated water	ref.			ref.		
	Well or natural water	1.65	1.23–2.22	0.001	1.87	1.38–2.53	<0.001
**Travel time to closest rural health unit or hospital**	<10 min	ref.					
	≥10 min	1.16	0.90–1.51	0.249			
**Travel time to Biliran Provincial Hospital**	<30 min	ref.					
	≥30 min	1.07	0.84–1.36	0.584			
**Municipality**	Almeria	ref.					
	Culaba	0.63		0.243			
	Biliran	1.27		0.462			
	Naval	1.34		0.278			
	Kawayan	1.23		0.488			
	Cabucgayan	1.69		0.067			
	Caibiran	1.59		0.102			

HR: Hazard ratio, 95% CI: 95% confident interval, socioeconomic status score: 1st quartile (41–92), 2nd quartile (27–40), 3rd quartile (16–26), and 4th quartile (0–15)

Educational system in the Philippines was preschool for three years (4–6 years old), elementary school for six years (7–12 years old), and secondary school for four years (13–16 years). With regard to the type of fuel used for cooking, others include charcoal, kerosene, electricity, and LP gas.

The Cox proportional hazards model was used. Variables with *p*-value of <0.1 in univariate analyses were included in a step-down procedure in multivariable models. The total socioeconomic status score and the individual factors included in the socioeconomic status score were tested in separated multivariate models. A history of asthma, the source of drinking water, or the geographic position of the house were missing in 5 episodes, 1 episode and 2 episodes, respectively; therefore, 264 episodes were included in multivariate analysis.

### History of asthma

Seventeen percent (866/5,225, 24 were missing data) of children who participated in this study had a history of asthma. Among those who did not experience pneumonia-like episodes, 14% (665/4,828) had a history of asthma. Among pneumonia-like episodes, 55% (256/462) had a history of asthma.

### Healthcare-seeking behavior

**Fig 3:**
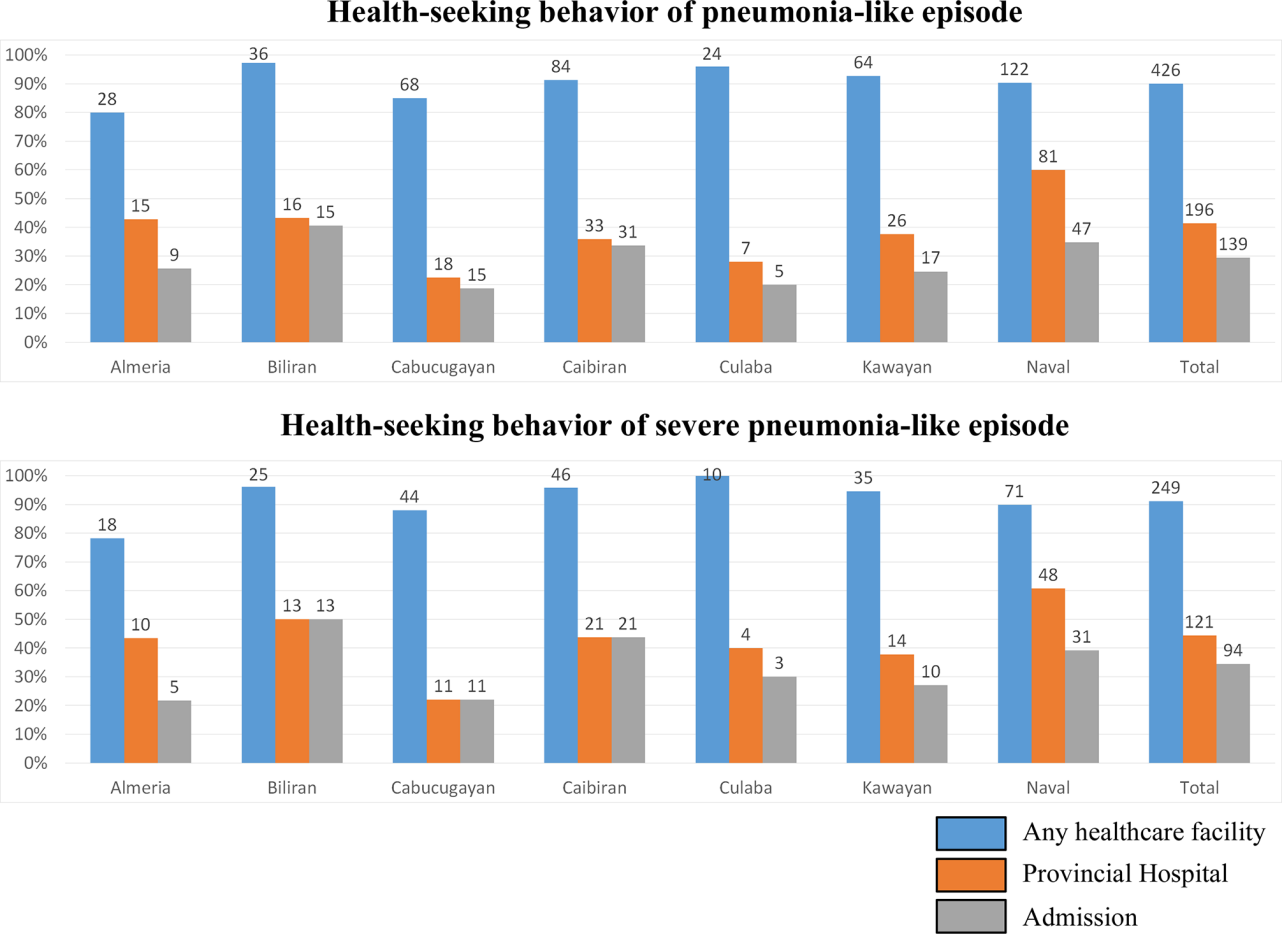
The rate of healthcare-seeking behavior of children who experienced a pneumonia-like episode according to the municipality, Biliran, the Philippines. The bars indicate the percentage and the numbers above the bars indicate actual counts. Any healthcare facility: Percentage of children who experienced a pneumonia-like episode and sought care at any healthcare facility: Barangay Health Workers, Barangay Captains, Barangay Health Stations, Rural Health Units, private clinics, or hospitals. Provincial hospital: Percentage of children who experienced a pneumonia-like episode and visited Biliran Provincial Hospital. Admission: Percentage of children who experienced a pneumonia-like episode and were admitted to Biliran Provincial Hospital.

Of 473 pneumonia-like episodes (one episode was excluded because of missing data), caregivers of 426 (90.0%) episodes reported having sought healthcare in at least one of the medical facilities (Fig 3). Among them, RHU was the most frequently visited facility (n = 243, 57.0%). One hundred ninety-six children (41.4%) visited a hospital during a pneumonia-like episode and 139 (29.3%) children were admitted. Among 273 severe pneumonia-like episodes, 249 (91.2%) visited any healthcare facility. One hundred twenty-one (44.3%) children visited a hospital and 94 (34.4%) children were admitted.

In univariate analysis, children with a longer travel time to the closest medical facility (OR: 0.50, 95% CI: 0.27–0.95) and a SES score in the second quartile compared with that in the first (highest) quartile (OR: 0.30, 0.10–0.85) were significantly less likely to seek healthcare in any medical facility when they experienced pneumonia-like episodes (Table 4). In addition, an older age (1 year old compared with 0 year old, OR: 0.46, 95% CI: 0.20–1.10), the presence of chest indrawing (OR: 1.89, 95% CI: 0.96–3.73), and the caregiver’s education level (OR: 1.94, 95% CI: 0.92–4.10) were selected for multivariate analysis (*p* < 0.1). In multivariate analysis, the SES score [second quartile compared with the first (highest), OR: 0.32, 0.11–0.95] remained significant and a longer travel time had borderline significance (OR: 0.52, 95% CI: 0.27–1.00, *p* = 0.050). In univariate analysis, children who experienced severe pneumonia episodes and had a household member with salaried employment (OR: 1.81, 95% CI: 1.04–3.15) tended to seek hospital care. In contrast, children with a longer travel time to the closest medical facility (OR: 0.48, 95% CI: 0.27–0.85) or to a hospital (OR: 0.32, 95% CI: 0.19–0.54) were less likely to seek hospital care. In multivariate analysis, the travel time to any medical facility (OR: 0.55, 95% CI: 0.31–0.98) and to the hospital remained significant (OR: 0.34, 95% CI: 0.20–0.58).

**Table 4:** Odds ratios of each factor for seeking any healthcare facilities among children less than 5 years of age who experienced pneumonia-like episodes (n = 473) or severe pneumonia-like episodes (n = 274) in Biliran, the Philippines.

	Healthcare-seeking behavior						
	Medical facility	Any healthcare facility			Biliran Provincial Hospital		
	Type of episode	Pneumonia-like episode			Severe pneumonia-like episode		
**Variables**		OR	95% CI	*p-*value	OR	95% CI	*p-*value
**Age of the child (in years)**	0	ref.			ref.		
	1	0.46	0.20–1.10	0.080	0.91	0.47–1.75	0.769
	2	0.59	0.22–1.58	0.296	0.88	0.39–1.99	0.767
	3	1.51	0.48–4.78	0.487	1.05	0.51–2.15	0.894
	4	0.67	0.23–1.94	0.464	1.00	0.47–2.10	0.996
**Sex**	Female	ref.			ref.		
	Male	1.17	0.62–2.21	0.619	0.61	0.37–1.02	0.058
**Unable to drink**	No	ref.			ref.		
	Yes	1.62	0.74–3.55	0.231	0.90	0.55–1.46	0.660
**Chest indrawing**	No	ref.			ref.		
	Yes	1.89	0.96–3.73	0.065	1.27	0.72–2.24	0.400
**Caregiver's education level**	<Secondary School						
	≥Secondary School	1.94	0.92–4.10	0.080	1.59	0.93–2.73	0.091
**History of asthma**	No	ref.			ref.		
	Yes	1.38	0.72–2.65	0.325	1.42	0.84–2.41	0.187
**Social economic status score**	1st quartile (highest)	ref.			ref.		
	2nd quartile	0.30	0.10–0.85	0.024	0.90	0.42–1.95	0.794
	3rd quartile	0.48	0.16–1.45	0.193	0.89	0.43–1.84	0.747
	4th quartile (lowest)	0.51	0.17–1.54	0.234	0.67	0.33–1.36	0.264
**Household member with salaried employment**	None	ref.			ref.		
	≥One	1.18	0.58–2.43	0.645	1.81	1.04–3.15	0.036
**Travel time to the closest rural health unit or hospital**	<10 min	ref.			ref.		
	≥10 min	0.50	0.27–0.95	0.034	0.48	0.27–0.85	0.011
**Travel time to Biliran Provincial Hospital**	<30 min	ref.			ref.		
	≥30 min	0.81	0.43–1.53	0.516	0.32	0.19–0.54	<0.001

OR: Odds ratio, socioeconomic status score: 1st quartile (41–92), 2nd quartile (27–40), 3rd quartile (16–26), and 4th quartile (0–15)

Educational system in the Philippines was preschool for three years (4–6 years old), elementary school for six years (7–12 years old), and secondary school for four years (13–16 years).

Odds ratios were calculated by generalized estimating equation for logistic regression to evaluate the factors associated with healthcare-seeking behaviors, including 1) visiting any healthcare facility when experiencing pneumonia-like episodes and 2) visiting a hospital when experiencing severe pneumonia-like episodes. Variables with missing data are a history of asthma (n = 12), the travel time to the closest RHU or hospital (n = 4), and the travel time to Biliran Provincial Hospital (n = 4) for #1 and a history of asthma (n = 7), the travel time to the closest RHU or hospital (n = 2), and the travel time to Biliran Provincial Hospital (n = 2) for #2.

**Fig 4:**
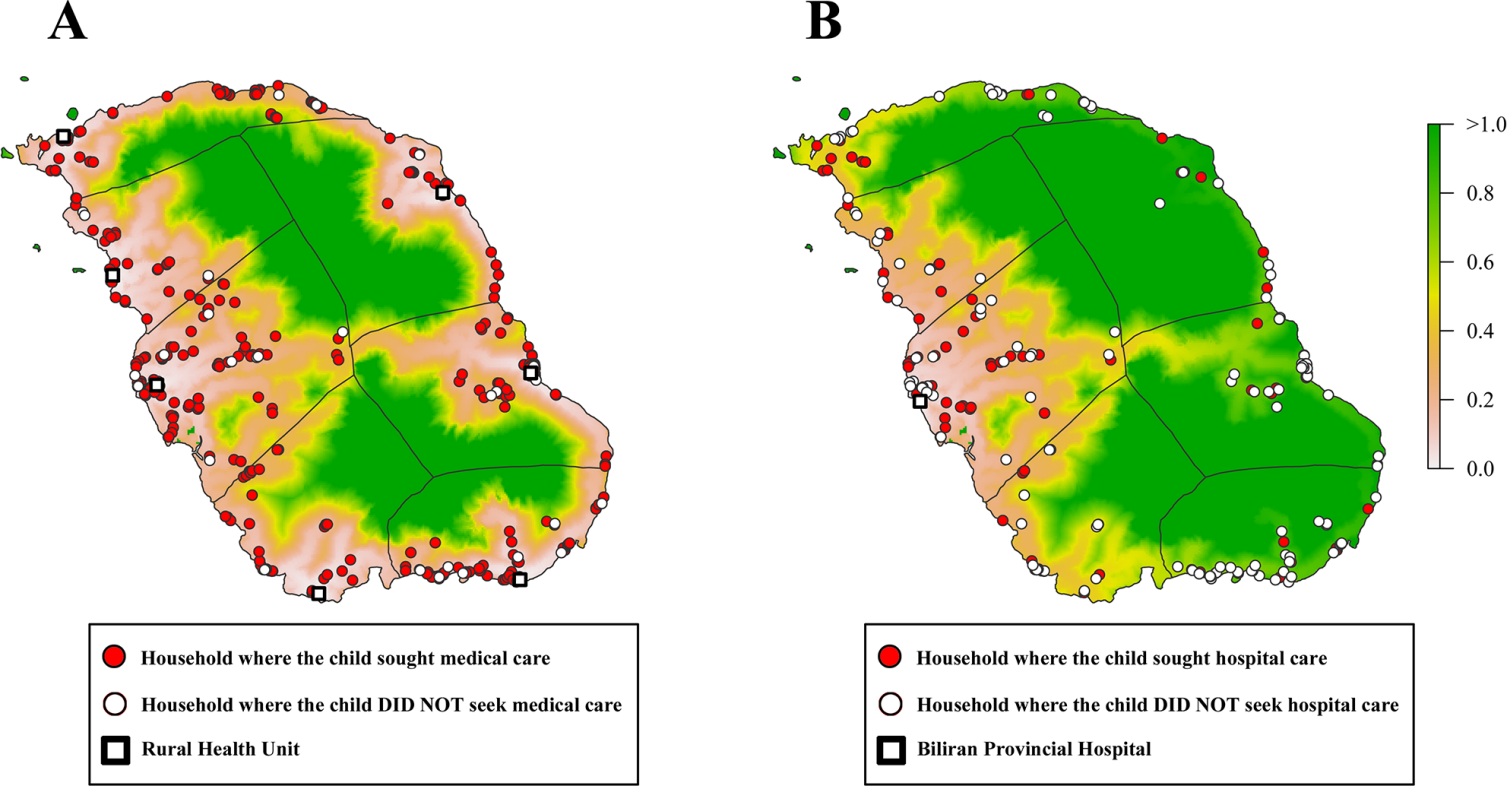
Healthcare-seeking behavior of children who experienced pneumonia-like episodes sought a primary healthcare facility or a hospital (A) and severe pneumonia-like episodes sought Biliran Provincial Hospital (B) by a household plot overlaid on the map of the travel time to healthcare facilities. The color scale shows the travel time (in hours) from each point of the map to the closest public healthcare facility (A) and to Biliran Provincial Hospital (B) estimated by the cost distance analysis.

The travel time and healthcare-seeking patterns are shown in maps (Fig 4). Because most of the children who experienced pneumonia-like episodes visited at least one healthcare facility, there appeared to be no particular geographic pattern of households with children who experienced pneumonia-like episodes, in which children did not seek care in any health facility (Fig 4A). However, for severe pneumonia-like episodes, apparent geographic differences were observed. Households with children who experienced severe pneumonia-like episodes, in which children did not seek hospital care were mainly seen in areas with a longer travel time to the hospital (Fig 4B). Naval, Almeria, and the western part of Kawayan had higher proportions of households seeking hospital care compared with eastern municipalities, in which the travel time to the hospital is longer.

### Pneumonia-associated mortality

Four children were suspected to have died from pneumonia during the study period (Table 5), resulting in a mortality rate from pediatric pneumonia-like episodes of 0.9 per 1,000 person-years. All four households of these children had lower SES scores compared with the mean score of all study households (mean: 31.6). Two of them (Cases No. 2 and No. 4) died outside the hospital, and the two others were from Culaba (Cases No. 3 and No. 4), which is the municipality that is farthest from BPH.

**Table 5:** Verbal autopsy data of four fatal cases who were suspected to have died of a pneumonia-like episode in Biliran, the Philippines, from June 2011 to May 2012.

	Case 1	Case 2	Case 3	Case 4
**Municipality**	Almeria	Biliran	Culaba	Culaba
**Sex**	Male	Male	Female	Male
**Age at death (in month)**	1	31	48	2
**Place of death**	Hospital	Home	Hospital	On the way to RHU
**Consulted any healthcare facility**	Yes	Yes	Yes	No
**Type of 1st healthcare facility**	Private clinic	RHU	RHU	N/A
**Hospital consultation**	Yes	No	Yes	No
**Admission**	Yes	No	Yes	No
**Chest indrawing**	No	Yes	No	No
**Difficulty in breathing**	Yes	No	Yes	Yes
**Fast breathing**	Yes	No	Yes	Yes
**Socioeconomic status score**	26	10	17	18
**Travel time to the closest rural health unit or hospital**	<10 min	<10 min	<10 min	<10 min
**Travel time to Biliran Provincial Hospital**	<30 min	<30 min	≥30 min	≥30 min

## Discussion

In this study, we estimated incidence rates of pneumonia-like episodes and severe pneumonia-like episodes and of pneumonia-associated mortality in Biliran Island. Risk factors associated with the occurrence of pneumonia-like episodes included a low SES, a history of asthma, the age of the child, a preterm birth, and the travel time to the healthcare facility. We also found that the strongest factor associated with healthcare-seeking behavior was the travel time to the healthcare facility.

Comparing demographic information of Biliran to that from Philippines National Demographic and Health Survey, children living in Biliran were less likely to have treated water (87.6 vs 95.2) and a water sealed toilet facility (57.5 vs 88.3) [27].

Our incidence rate of 105 pneumonia-like episodes per 1,000 person-years for children less than 5 years of age was similar to that of pneumonia in children less than 5 years of age in the Western Pacific region (110 per 1,000 person-years) [3] and the incidence rate of 61 severe pneumonia-like episodes per 1,000 person-years was similar to that of acute lower respiratory infection (ALRI) in Thailand (57.7 per 1,000 person-years) [28]. However, our incidence rate of pneumonia-like episodes was higher than that reported in previous studies on pneumonia conducted at hospital settings in the Philippines (at Bohol), Brazil, or Uruguay [8,29,30] and lower than that reported in a community-based cohort study conducted in Bangladesh, in which the ALRI incidence rate in children was 320 per 1,000 person-years [31]. The incidence rates of pneumonia can differ depending on clinical criteria, the inclusion of radiographic confirmation, the hospital or community-based setting, and the person who makes diagnosis. For example, a study conducted in Kenya demonstrated that the sensitivity of detecting child pneumonia at a hospital is likely to be 45% less than that of detecting pneumonia in children at the community, because some pneumonia patients may not visit a hospital [32]. In a clinical trial study of a pneumococcal conjugate vaccine in Bohol, Philippines, the incidence rate of clinical pneumonia among children aged less than 2 years in the placebo group was 105 per 1,000 person-years (in the vaccine group it was 104 per 1,000 person-years) [30], which was lower than that observed in our study (139 per 1,000 person-years for children less than 2 years of age). The Bohol study included pneumonia cases identified by pediatricians at the hospital, and tachypnea was among the clinical criteria but fever was not. The annual number of pediatric pneumonia cases estimated in our study was comparable to that provided by the Field Health Service Information System of the National Epidemiology Center (1,852 cases in Biliran Province, 2011) [33].

The diagnosis of pneumonia, especially differentiating it from asthma or bronchitis, is challenging in children less than 5 years of age, even in a hospital setting [34–36]. The diagnosis of asthma using clinical criteria is also difficult in children less than 5 years of age, especially in infants [37]. In our criteria for pneumonia-like episodes, there is a possibility of the misclassification of an asthma attack or asthmatic bronchitis based on the caregiver’s recollection.

In the risk factor analysis, there was a strong association between pneumonia-like episodes and a history of asthma. Other studies have also reported that a history of asthma is associated with the occurrence of pneumonia episodes [38–41]. In a study conducted in Brazil, 53.8% of children who developed consolidated pneumonia had asthma as an underlying illness [42]. This proportion is similar to that of children in our study (55.4%) with a history of asthma who experienced pneumonia-like episodes. However from these results, we were unable to conclude whether uncontrolled asthma or an underlying asthmatic condition was a risk factor of pneumonia or that pneumonia was a trigger of asthma attack. This question has been discussed extensively in previous studies and remains to be solved [34,35,38–41]. However, misclassification between pneumonia-like episodes and asthma and recalling bias due to the retrospective nature of the study might have an impact on the association between pneumonia and asthma since caregivers of asthmatic children remembered the pneumonia episode more than those with non-asthmatic children. A prospective cohort study is required to define a causal relationship between asthma and pneumonia.

A lower SES was also associated with the occurrence of a pneumonia-like episode, as indicated in previous studies in other countries [5–7,9,43–45]. It has been shown that a low SES makes children more susceptible to pneumonia [46,47]. Because we included all pneumonia-like episodes occurring in the community, our analysis of the association between pneumonia-like episodes and socioeconomic factors might be more accurate than most previous studies, in which data were collected only from hospital settings and did not include patients who did not seek hospital care [6,44,47].

In a previous study, age was found to be associated with the occurrence of pneumonia-like episodes [47]. In our study, preterm gestational age at birth was also observed as one of the significant risk factors. However, in the present study, other risk factors such as parental smoking, number of siblings, and indoor air pollution, which have been shown to be risk factors for childhood pneumonia or acute lower respiratory tract infection, were not significantly associated with the occurrence of pneumonia-like episodes [6,43–45,48].

A longer travel time to the closest healthcare facility was a significant risk factor for experiencing a pneumonia-like episode. After adjusting for demographic and socioeconomic factors, the travel time remained significant. This result indicates that a greater distance from the healthcare facility may impede appropriate healthcare and contribute to the deterioration of acute respiratory infection. A strong negative association between healthcare-seeking behavior and a longer travel time was also reported in previous studies [19,32]. Compared with SES, the travel time had a stronger effect on healthcare-seeking behavior.

In our study, most of the children sought care at a healthcare facility, but only 44% of these children sought care at a hospital during severe pneumonia-like episodes. We observed a significant impeding effect of the travel time to seek hospital care among children who experienced severe pneumonia-like episodes, although most of the children could travel to the hospital within 1 hour, as defined by cost distance. A study conducted in Estonia demonstrated a substantial decrease in hospitalizations of the population if the travel time to the hospital was more than 30 min [49]. Another study in Kenya also demonstrated that the hospitalization rate decreased over 20% per hour of travel time to the hospital [32]. Further investigation is required in order to understand why children living in far places do not visit hospitals often although they have sought care at a healthcare facility on at least one occasion.

We found four fatal cases due to pneumonia-like episodes during the study period. On the other hand, the number of pneumonia-related deaths based on BPH records was 11, and the mortality was 0.6 per 1,000 person-years (personal communication, hospital/other health facilities statistical report, 2011–2012, DOH, Philippines). Mortality rate due to pneumonia-like episodes in our study was higher (0.9 per 1,000 person-years) than that reported by the BPH described above. It should be noted that two of four cases died outside the hospital. Because of the small number of fatal cases, it was difficult to evaluate the effect of deaths outside the hospital on the overall mortality rate. It was also not possible to evaluate risk factors for these fatal cases.

## Conclusion

One of ten children less than the age of 5 years develops pneumonia-like episodes per year in Biliran Island, Philippines. Risk factors, including the age of a child, the gestational age at birth, a history of asthma, SES, and the travel time to a healthcare facility, were significantly associated with the occurrence of pneumonia-like episodes, but the causality between pneumonia development and risk factors such as a history of asthma remains elusive. With regard to healthcare-seeking behavior, the travel time was observed as a more important factor than the other factors, including SES. To improve healthcare-seeking behavior, better access to healthcare facilities may be a key determinant, and they would also aid early and effective management of patients with pneumonia. Further longitudinal studies are warranted for a better understanding of these findings.

## Supporting Information

S1 Table Cost assignment of each land cover and road. The slope cost was defined by the Tobler's hiking function using the walking speed on the land cover map.
(DOCX)

S1 Fig The number of children who experienced pneumonia-like episodes (A) and severe pneumonia-like episodes (B) per a household overlaid on the map of the travel time to healthcare facilities. The color scale indicates the travel time (in hours) from each point of the map to a closest public healthcare facility (A) and to Biliran Provincial Hospital (B) estimated by the cost distance analysis.
(TIF)

S1 Dataset Person-year and data. (XLSX)

S2 Dataset Longitudinal pneumonia episode data. (XLSX)

S3 Dataset Health care seeking data. (XLSX)
