# Supplementary material for: Incidence and Risk Factors of Childhood Pneumonia-Like Episodes in Biliran Island, Philippines—A Community-Based Study
Source: PLoS One. 2015 May 4;10(5):e0125009. doi: 10.1371/journal.pone.0125009 (PMC4418693; doi:10.1371/journal.pone.0125009)
Supplement: S1 Table — The slope cost was defined by the Tobler's hiking function using the walking speed on the land cover map. (DOCX) [file pone.0125009.s001.docx]

**S1_ Table. Cost assignment of each land cover and road.**

| Type of land | Speed (km/h) | METHOD |
| --- | --- | --- |
| Bare areas | 5 | Walking |
| Urban | 5 | Walking |
| Low dense vegetation | 4 | Walking |
| Medium dense vegetation | 3 | Walking |
| Dense vegetation | 2 | Walking |
| Mangrove forest | 9 | Boating |
| Peripheral secondary road | 40 | Motorcycle |
| Tertiary and other Secondary road | 30 | Motorcycle |
| Track and Residential road | 20 | Motorcycle |

The slope cost was defined by the Tobler's hiking function using walking speed on the land cover map.
