# Supplementary figures and images for: Incidence and Risk Factors of Childhood Pneumonia-Like Episodes in Biliran Island, Philippines—A Community-Based Study
Source: PLoS One. 2015 May 4;10(5):e0125009. doi: 10.1371/journal.pone.0125009 (PMC4418693; doi:10.1371/journal.pone.0125009)

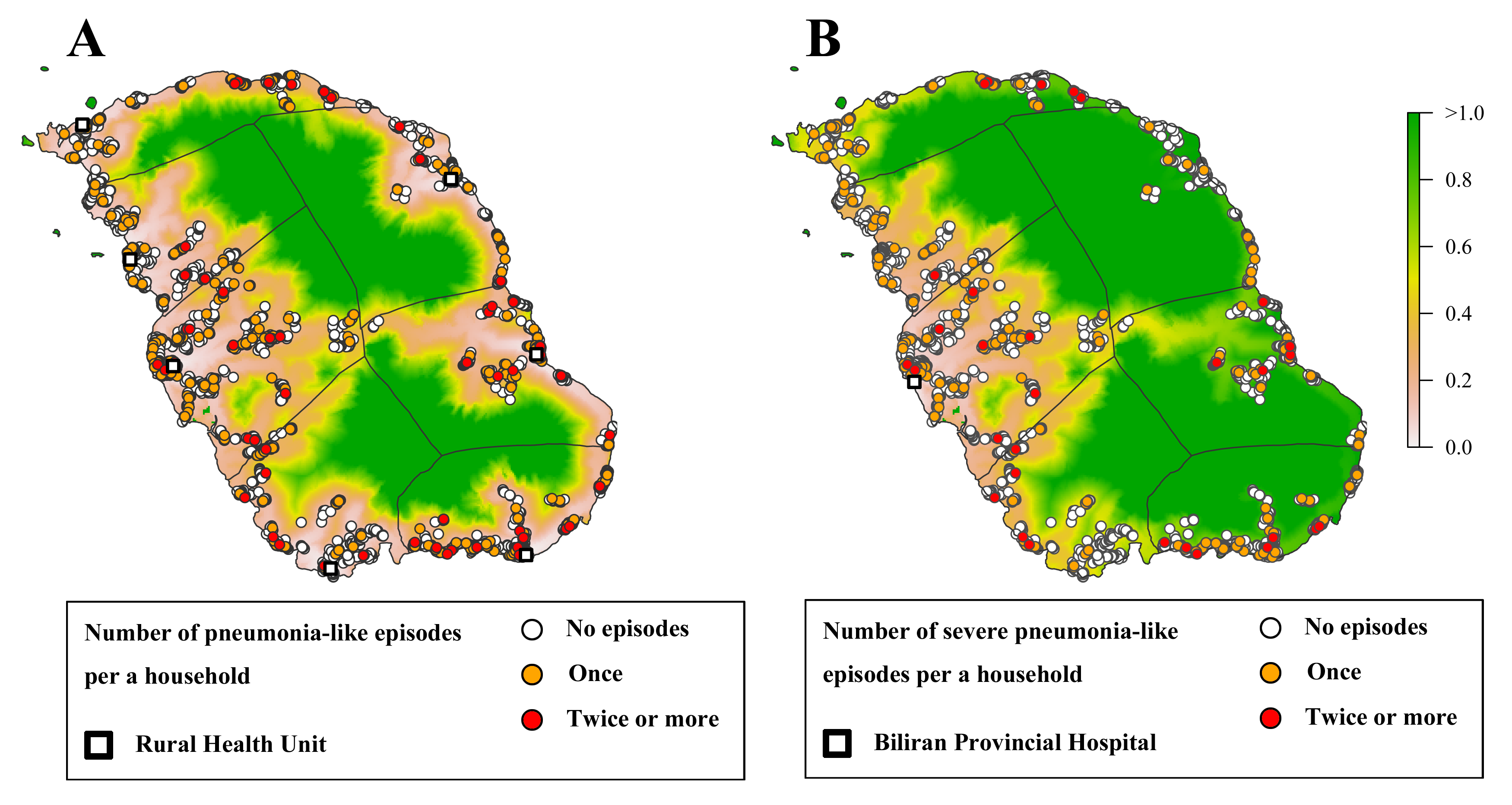

Supplement: S1 Fig — The color scale indicates the travel time (in hours) from each point of the map to a closest public healthcare facility (A) and to Biliran Provincial Hospital (B) estimated by the cost distance analysis. (TIF) [file pone.0125009.s002.tif]
